# Pivotal Role of Mediterranean Dietary Regimen in the Increase of Serum Magnesium Concentration in Patients with Coronary Artery Disease

**DOI:** 10.1155/2013/431070

**Published:** 2013-11-07

**Authors:** Nimah Bahreini, Mojgan Gharipour, Hossein Khosravi-Boroujeni, Hojjat Rouhi-Boroujeni, Afshin Shiranian, Amin Salehi-Abargouei, GholamReza Sharifzadeh

**Affiliations:** ^1^Food Security Research Center, Department of Community Nutrition, School of Nutrition and Food Science, Isfahan University of Medical Sciences, Isfahan, Iran; ^2^Department of Metabolic Syndrome, Isfahan Cardiovascular Research Center, Isfahan Cardiovascular Research Institute, Isfahan University of Medical Sciences, Isfahan, Iran; ^3^Medical Plants Research Center, Shahrekord University of Medical Sciences, Shahrekord, Iran; ^4^Health Research Centers of School of Health, Birjand University of Medical Sciences, Birjand, Iran

## Abstract

*Background*. Recent studies confirmed cardioprotective role of intravenous magnesium for the prevention of cardiac events, but effect of dietary intake of this mineral via recommended dietary regimens on control and inhibition of coronary artery disease (CAD) risk factors has been questioned. The aim of the present study was to determine effect of Mediterranean dietary approach on serum magnesium concentration among Iranian patients with CAD. *Method*. Baseline characteristics and clinical data of 102 consecutive patients with the diagnosis of CAD and candidates for isolated coronary artery bypass surgery were entered into the study. Laboratory parameters especially serum magnesium concentration were measured after 12–14 h of overnight fasting and before operation. Nutritional status was assessed by food frequency questionnaire and the diet score was calculated on the basis of Mediterranean diet quality index (Med-DQI). *Results*. No significant differences were found in the concentrations of albumin, last fasting blood sugar, last creatinine, and lipid profiles between the groups with Mediterranean dietary score < 5 and the group with higher dietary score; however, serum magnesium concentration in the first group was higher than that in the group with higher dietary score. Linear multivariate regression analysis showed that the lower Mediterranean dietary score was a predictor for serum magnesium concentration after adjusting for confounders. *Conclusion*. Taking Mediterranean dietary regimen can be associated with increased level of serum magnesium concentration, and thus this regimen can be cardioprotective because of its effects on serum magnesium.

## 1. Introduction

The dietary magnesium intake tends to be lower than that recommended worldwide even in western countries. Several factors have been identified to be trigger for lowering magnesium intake including the waterborne magnesium factor, the loss of magnesium during food refining, and the magnesium content of vegetarian diets, as well as various metabolic situations such as hypertension, pregnancy, osteoporosis, drug therapy, alcoholism, stress, and even cardiac trauma. Magnesium is the most abundant intracellular divalent cation that is important for physiological processes including neuromuscular function and maintenance of cardiovascular tone. 

The importance of magnesium intake in relation to cardiovascular diseases has been increasingly described, so that its intake can be inversely related to the risk of hypertension and type 2 diabetes mellitus [1] and may improve serum lipid profiles [2]. Recent surveys in Sweden [3, 4] have indicated that vegetarian diets provide a more adequate intake of magnesium, as illustrated in Table 4. Note that the magnesium intake of men and women on vegetarian diets was considerably greater than the RDA requirement; this was attributed to the high intake of vegetables and whole-grain cereals by these individuals. Recent studies have shown that some components of the Mediterranean diet such as vegetables, legumes, and nuts are rich sources of magnesium; therefore, it seems that the high adherence to the Mediterranean diet is associated with high consumption of magnesium [5]. Although the main dietary regimen of Iranian population is Mediterranean diet components especially the major sources of magnesium, the rate of coronary artery disease (CAD) risk factors among this population has been considerably high [6]. Therefore, in the present study, we tried to determine effect of Mediterranean dietary approach on laboratory parameters related to CAD risk factors with the focus on serum magnesium concentration among Iranian patients with CAD. 

## 2. Methods

### 2.1. Study Population

Baseline characteristics and clinical data of 102 consecutive patients with the diagnosis of CAD and candidates for isolated coronary artery bypass surgery were entered into the study. Studied data were collected by the review of clinical recorded files in the day of admission. In this study, CAD was considered significant if there was a 75% or greater stenosis in the cross-sectional diameter and 50% or greater stenosis in the luminal view [7]. 

### 2.2. Laboratory Measurements

Blood samples were drawn from the corresponding peripheral vein into vacutainer tubes after 12–14 h of overnight fasting and before operation. Plasma glucose concentrations were assessed by means of a glucose hexokinase method (Pars Azmoon kits accredited by Bioactiva Diagnostica, Germany). Serum total cholesterol, HDL cholesterol, and triglycerides were measured via enzymatic techniques (Pars Azmoon kits accredited by BioactivaDiagnostica, Germany). The Friedewald formula was used to calculate low density lipoprotein (LDL) cholesterol, except when the triglyceride level was >4.52 mmol/L. Blood pressure was calculated as the mean of two measurements, performed in the sitting position after 5 minutes of rest, using a random-zero sphygmomanometer (Hawksley-Gelman, Lancing, Sussex, UK). C-reactive protein (CRP) level was measured by immunoturbidimetry (Pars Azmun, Iran), and lipoprotein(a) was measured using Tint ELIZA (Biopool, USA). Creatinine was measured with Jaffe reaction (Parsazmon Kit, Tehran, Iran) using an autoanalyzer (Hitachi, Tokyo, Japan). Serum magnesium level was measured calorimetrically by an autoanalyzer. 

### 2.3. Mediterranean Diet

Studied patients were also interviewed on admission to surgical ward and before operation to report the consumption of food items listed as the number of times per day, per month, or per year during the previous year. We assessed nutritional status by a validated food frequency questionnaire (FFQ), previously validated in Iran [8] and a 24-hour dietary recall questionnaire to record the types, amounts, and frequencies of foods consumed. Sum of the consumption of each of several food items was used to determine the overall consumption of the food group to which each item belonged [9, 10]. The diet score was calculated on the basis of Mediterranean diet quality index (Med-DQI) that the construction of the score for this Index (Table 1). The index assigns a score of 0, 1, or 2 according to the daily intake of each of the seven components and then final score was reported as a summation of all nutrient scores ranged between 0 and 14. A lower score on this index indicates a better nutrition quality [11]. We divided studied patients into two groups according to the final score: the groups with final score lower than 5 (*n* = 35) and those who had final score between 5 and 10 (*n* = 67). None of the patients had final score more than 10.

### 2.4. Statistical Analysis

We compared categorized variables between the two above groups using Chi-square test or Fisher's exact test if required. Continuous variables were also compared using independent *t*-test. Multivariate linear regression analysis was used to investigate the potential confounding effects of patients' characteristics and clinical data on the association between serum magnesium concentration and Mediterranean regimen score. *P* values of 0.05 or less were considered statistically significant. All the statistical analyses were performed using SPSS version 13.0 (SPSS Inc., Chicago, IL, USA).

## 3. Results

Studied patients had the mean age of 58.8 year (ranged between 38 and 78 years) and 40.2% of them were male. Hyperlipidemia and hypertension were observed in 75.5% and 56.9% of patients, respectively, and 51.0% of them were diabetics. Half of the studied patients had moderate functional class, but three coronary arteries were involved in the majority of them (Table 2). Comparison of the concentrations of serum laboratory parameters between the two groups with lower Mediterranean dietary score and group with higher dietary score showed no significant differences between the concentration of albumin, last fasting blood sugar, last creatinine, and lipid profiles (Table 3); however, serum magnesium concentration in the group with dietary score between 5 and 10 was lower than that in group with higher score (*P* = 0.026). Linear multivariate regression analysis also showed that the lower Mediterranean dietary score was a predictor for serum magnesium concentration after adjusting for confounders (Table 4). 

## 4. Discussion

Recent studies could confirm the cardioprotective role of intravenous magnesium for prevention of cardiac arrhythmia, pump dysfunction, and death in patients with CAD, especially in the immediate postinfarction period [12, 13]. The mechanism of this role has been also described. Magnesium can inhibit the influx of calcium in vascular smooth muscle cells and therefore inhibit arrhythmic recurrence and the production of IL-6 and MMP-1 after reperfusion and prevent the increase of myocardial lesions caused by calcium overload on myocytes [14]. However, effect of dietary intake of this mineral on control and inhibition of CAD risk factors has been questioned. Some researchers have been hypnotized that the low intake of magnesium may be related to the least degree of cardiovascular disease [15, 16]. Some others could show that the dietary magnesium intake can be inversely associated with fasting serum insulin, plasma high density lipoprotein-cholesterol, and systolic and diastolic blood pressure [17].

In the present study, we showed the potential effect of Mediterranean dietary regimen on increasing of serum magnesium concentration that can mainly lead to the favorite control and prevention of CAD risk factors. We have shown that not only intravenous magnesium administration can be related to the lower risk of poor outcome in patients with CAD, but also these effects can be obtained by dietary intake of this mineral via Mediterranean dietary regimen. In a similar study by Schröder, it was found that the high consumption of vegetables, fruits, legumes, nuts, fish, cereals, and olive oil as main components of Mediterranean regimen led to high absorption of magnesiumin obesity and type 2 diabetes [18]. Also, Singh showed that the increased intake of dietary magnesium in association with the general effects of a nutritious diet can offer protection against cardiovascular deaths among high-risk individuals predisposed to CAD [19]. Furthermore, in another study, it was observed that the intake of dietary magnesium was associated with a reduced risk of CAD; however, associations between dietary magnesium and coronary events occurring after fifteen years of follow-up were modest [20]. It seems that the Mediterranean dietary regimen and its components as rich pools of magnesiummight optimize micronutrient status in main body organs especially cardiovascular system [21]. 

In the present study, it was also found that the patients' baseline characteristics and even severity of CAD had no relationship with serum magnesium concentration in multivariate analysis. Similarly, in a study by Mataix et al., the risk of hypomagnesemia was not associated with any of the other factors that were investigated such as gender, educational level, obesity, smoking habits, alcohol consumption, and physical exercise [22]. However, some studies showed that the magnesium intake could decrease in advanced age and in men for each of the different race or ethnic groups [23]. It seems that the serum magnesium changes are mainly dependant on nutritional habit, metabolicprocesses, inflammatory biomarkers, and other related-potential mechanisms that should be investigated in future studies. 

In conclusion, taking Mediterranean dietary regimen can be associated with increased level of serum magnesium concentration, and thus this regimen can be cardioprotective because of its effects on serum magnesium.

## Figures and Tables

**Table 1:** Construction of the score for the Mediterranean dietary quality index (lower score indicates higher quality of regimen).

Scoring	0	1	2
Saturated fatty acids, % energy	<10	10–13	>13
Cholesterol, mg	<300	300–400	>400
Meats, g	<25	25–125	>125
Olive oil, mL	>15	15–5	<5
Fish, g	>60	60–30	<30
Cereals, g	>300	300–100	<100
Vegetables + fruits, g	>700	700–400	<400

**Table 2:** Demographic characteristics and clinical data of studied patients (*n* = 102).

Male gender	41 (40.2)
Age (year)	58.86 ± 8.90
Body mass index (kg/m^2^)	32.57 ± 2.46
Family history of CAD	53 (52.0)
Current cigarette smoking	30 (29.4)
Opium addiction	9 (8.8)
Diabetes mellitus	
Hyperlipidemia	77 (75.5)
Hypertension	58 (56.9)
Cerebrovascular disease	7 (6.9)
Peripheral vascular disease	35 (34.3)
Recent myocardial infarction	49 (48.0)
Renal failure	11 (10.8)
Ejection fraction (%)	50.00 ± 9.59
Functional class:	
I	26 (25.5)
II	51 (50.0)
III	25 (24.5)
Euroscore	2.25 ± 2.06
Coronary vessels involvement:	
Single-vessel disease	3 (3.0)
Two-vessel disease	24 (23.5)
Three-vessel disease	75 (73.5)

CAD: coronary artery disease.

**Table 3:** Laboratory parameters in the groups with the dietary score lower than 5 and group with higher dietary score.

Laboratory parameters	Dietary score lower than 5 (*n* = 35)	Dietary score between 5 and 10 (*n* = 67)	*P* value
Magnesium	2.01 ± 0.40	1.80 ± 0.28	0.026
Last fasting blood sugar (mg/dL)	111.38 ± 30.25	109.43 ± 31.83	0.763
Last creatinine (mg/dL)	1.21 ± 0.28	1.25 ± 0.24	0.498
Hematocrit	41.18 ± 4.02	41.75 ± 4.34	0.510
Triglyceride (mg/dL)	175.92 ± 58.09	178.49 ± 94.67	0.866
Cholesterol (mg/dL)	168.24 ± 43.73	161.14 ± 42.49	0.435
High density lipoprotein (mg/dL)	41.64 ± 9.29	40.43 ± 8.62	0.525
Low density lipoprotein (mg/dL)	89.94 ± 37.19	85.79 ± 33.98	0.584
Lipoprotein (a) (mg/dL)	36.00 ± 28.25	27.92 ± 27.46	0.172
Albumin (g/dL)	4.65 ± 0.26	4.65 ± 0.27	0.918
C-reactive protein	6.33 ± 1.70	6.31 ± 4.58	0.974

**Table 4:** Multivariate analysis for determining the effects of Mediterranean dietary score on serum magnesium concentration adjusted for baseline variables as confounders.

Variables	Univariate *P* value	Multivariate *P* value	Multivariate analysis	
			Standardized beta	95% CI for beta
Mediterranean dietary score	0.026	0.022	−0.289	−0.032–0.399
Male gender	0.037	0.116	0.207	−0.039–0.345
Number of defected coronary vessels	0.044	0.944	−0.009	−0.181–0.169
Body mass index	0.065	0.165	−0.193	−0.078–0.014
C-reactive protein	0.071	0.027	0.280	0.006–0.096
Low density lipoprotein	0.021	0.031	0.276	0.000–0.005
